# Aligning Sleep Patterns with Educational Schedules: Policy Recommendations for Enhancing Health and Academic Success

**DOI:** 10.12688/f1000research.173727.1

**Published:** 2025-12-03

**Authors:** Ali Zakiei, Arash Ziapour, Mohammad-Taher Moradi, Habibolah Khazaie

**Affiliations:** 1 Sleep Disorders Research Center, Health Policy and Promotion Institute, Kermanshah University of Medical Sciences, Kermanshah, Kermanshah Province, Iran; 2 Cardiovascular Research Center, Health Policy and Promotion Institute, Kermanshah University of Medical Sciences, Kermanshah, Kermanshah Province, Iran

**Keywords:** Sleep, Education, Children, Adolescent Health, School Start Times, Circadian Rhythms, Academic Performance, Policy Analysis

## Abstract

**Background:**

This policy brief examines the significant misalignment between the biological sleep patterns of students and the early start times of most educational institutions. Chronic sleep deprivation in this population is associated with detrimental effects on academic performance, mental and physical health, and interpersonal relationships.

**Policy and implications:**

Compelling evidence indicates that early school schedules, dictated by historical and economic factors rather than student biology, force adolescents and young adults into a state of chronic sleep deficit. This has clear impacts, including impaired cognitive function, increased risks of obesity and cardiometabolic disease, and academic burnout. A systematic review of the literature confirms that delaying start times is an effective intervention to reduce these problems.

**Recommendations:**

This brief recommends systematic changes, including later school and university start times, the integration of sleep hygiene education into curricula, limits on late-evening activities, and the use of flexible learning models. Supporting these structural changes, systems for monitoring student well-being and educating families are also advised.

**Conclusions:**

Aligning educational schedules with biological sleep needs is a critical public health intervention. Comprehensive strategies that combine schedule adjustments, education, and support services offer the greatest benefit for student health and academic success. Future research should focus on socio-economic factors and local structural barriers to ensure equitable implementation.

## Introduction

Sleep is recognized as a vital and structured process that follows a regular, cyclical pattern each night. This organized sequence of events ensures the optimal restoration of both physical and mental functions. ^1^ Indeed, sleep plays a crucial role in human psychological and physical health and is a key factor in regulating emotion, cognition, psychosocial development, and physical growth. ^2^ Inappropriate sleep patterns constitute a serious risk factor for poor physical health ^3^ and significantly diminish quality of life. ^4^ It is essential for individuals to maintain proper sleep schedules and obtain sufficient sleep, as sleep insufficiency impairs daily functioning and incurs substantial human, social, and economic costs. ^5^ Since sleep timing is a critical aspect, ^6^ sleep should occur within an appropriate temporal framework.

Healthy sleep necessitates several conditions, including adequate duration, satisfactory quality, appropriate timing, and consistent regularity, and is also dependent on the absence of sleep disorders or anomalies. ^7^ However, healthy sleep, or sleep hygiene, receives insufficient attention today, and school and university schedules often disregard student sleep patterns in their educational planning. The scale of the issue is significant, with studies indicating that a considerable portion of the student population exhibits poor sleep hygiene and insufficient sleep quantity. ^8^ Consequently, this policy brief synthesizes current scientific understanding of how school and university schedules impact sleep timing and duration and provides evidence-based recommendations for curriculum planning and policy adjustments to improve student well-being and academic achievement.

## Policy outcomes and implications

This policy brief is based on a synthesis and analysis of the current scientific literature, including systematic reviews and key observational studies, to evaluate the impact of educational schedules on student sleep and health. A fundamental conflict exists between the rigid schedules of educational institutions and the biological clocks of students. Despite compelling evidence on adolescent sleep needs, school start times are often dictated by history and logistics, not student physiology. This conflict is exacerbated by modern lifestyles that push bedtimes later, while long commutes and early class times force students to wake prematurely. This misalignment between educational schedules and circadian rhythms exposes students to the well-documented consequences of chronic sleep deprivation, thereby putting at risk their health, safety, and academic performance. Research has shown that sleep problems during the educational years are linked to broader psychosocial challenges, underscoring the multifaceted impact of sleep on student well-being. ^9^

The core problem lies in the direct impact of early start times on sleep duration and quality. Early classes force students to wake earlier, which, even with unchanged bedtimes, truncates total sleep time and leads to a cumulative sleep deficit. This forced sleep restriction is associated with significant issues, including academic burnout and declined academic performance. ^10, 11^ The consequences, however, extend far beyond the classroom, demonstrating measurable physiological consequences. Inadequate sleep is linked to poorer diet quality, decreased insulin sensitivity, and hyperglycemia. ^12^ The scale of this impact is staggering. In adolescents, very short sleep can increase the odds of obesity by 69% and elevated waist circumference by 49%. ^13^ This significantly elevates their long-term risk for type 2 diabetes and cardiovascular disease. While these correlational data are compelling, we must also consider confounding factors like academic stress and familial pressures.

Revising school start times is therefore an evidence-based necessity, not just an optional adjustment. It is crucial for protecting student well-being and educational productivity. The core of the issue is chronobiological: when early schedules conflict with students’ innate circadian rhythms, the resulting sleep deficit directly undermines their academic performance, mental health, and behavior. Empirical evidence demonstrates the solution. Results from a systematic review indicated that delaying school start times significantly increases students’ nightly sleep duration by at least 30 minutes, primarily due to a later wake-up time. ^14^ Also, this policy change is associated with improved school attendance, reduced tardiness, decreased daytime sleepiness, and enhanced academic performance. ^14^ These findings highlight the need to reevaluate school timing policies. It is now widely argued that delayed start times allow students to experience more and better-quality sleep, ^15^ leading to reduced daytime sleepiness, improved cognitive and academic performance, ^16, 17^ and better overall physical and mental health. Successful implementation, however, requires a multi-systemic approach that considers potential conflicts with parental work schedules and the pervasive culture of intensive extracurricular commitments. In general, flexibility in the educational schedule could be proposed as a practical and acceptable alternative to a fixed later start time, provided that students are encouraged to adhere to a consistent sleep schedule. ^18^

## Actionable recommendations

Based on the analysis of policy outcomes and implications, we propose the following coordinated actions for educational policymakers and institutions. First, schools and universities should systematically their start times delay to better align with the circadian rhythms of adolescents and young adults, helping longer and more continuous sleep. As a foundational step, the Ministry of Education should issue guidelines mandating that classes not begin before 8:30 AM. Extracurricular and academic activities should be scheduled to conclude by 9:00-10:00 PM to prevent the significant delay in sleep onset and the reduction in total sleep duration that often follows late-evening commitments. Alongside these structural changes, integrating sleep hygiene education into core curricula is essential to raise awareness about the importance of sleep and to promote healthy sleep behaviors. This education should include specific, practical strategies for managing digital device use before bedtime and could be tailored to address individual factors, such as personality traits, that are known to influence adherence to sleep hygiene practices. ^8^ To support these efforts, systems for monitoring student sleep patterns and well-being should be established to allow for counseling and early intervention for those at risk of sleep deprivation or disorders. The educational message should promote healthy sleep patterns by emphasizing that sleep regularity, alongside duration, is critical for academic success and general health. Students and faculty should be informed about the benefits of maintaining a consistent sleep-wake schedule across the entire week, including weekends, to stabilize circadian rhythms and mitigate the effects of social jetlag. Additionally, we must educate families to guide lifestyle modifications that support healthy sleep at home. Finally, schools should create inherent system flexibility by exploring blended or online learning models. These formats can grant students autonomy over their schedules, allowing them to prioritize sleep without compromising academic responsibilities.

### Limitations of the analysis

While our recommendations are grounded in evidence, we acknowledge their reliance on observational studies, which show correlation rather than proven causation. Future research must also address the limited data within specific socio-cultural contexts, such as Iran, to ensure these policies are equitably implemented.

## Conclusions/Discussion

Aligning educational schedules with student sleep biology is a critical public health intervention. Thoughtfully planned curricula and supportive policies can significantly improve students’ mental sharpness, overall health, and academic performance. To achieve this, the body clocks of adolescents and young adults must be a primary factor in setting school start times and daily schedules. This alignment serves not only to boost immediate academic performance and mental health but also to help mitigate the long-term risk of cardiometabolic diseases associated with chronic sleep deprivation. The most substantial benefits for student health and academic performance will likely arise from comprehensive approaches that integrate schedule adjustments, targeted education, and robust student support services.

## Data Availability

No data are associated with this article.
