# Aligning Sleep Patterns with Educational Schedules in Iran: Policy Recommendations for Enhancing Health and Academic Success

**DOI:** 10.12688/f1000research.173727.2

**Published:** 2026-03-02

**Authors:** Ali Zakiei, Arash Ziapour, Mohammad-Taher Moradi, Habibolah Khazaie

**Affiliations:** 1 Sleep Disorders Research Center, Health Policy and Promotion Institute, Kermanshah University of Medical Sciences, Kermanshah, Kermanshah Province, Iran; 2 Cardiovascular Research Center, Health Policy and Promotion Institute, Kermanshah University of Medical Sciences, Kermanshah, Kermanshah Province, Iran

**Keywords:** Sleep, Education, Children, Adolescent Health, School Start Times, Circadian Rhythms, Academic Performance, Policy Analysis

## Abstract

**Background:**

This policy brief examines the significant misalignment between the biological sleep patterns of students and the early start times of most educational institutions, specifically within the context of the Iranian educational system. Chronic sleep deprivation in this population is associated with detrimental effects on academic performance, mental and physical health, and interpersonal relationships.

**Policy and implications:**

Compelling evidence indicates that early school schedules, dictated by historical and economic factors rather than student biology, force adolescents and young adults into a state of chronic sleep deficit. This has clear impacts, including impaired cognitive function, increased risks of obesity and cardiometabolic disease, and academic burnout. Studies confirm that delaying start times is an effective intervention to reduce these problems.

**Recommendations:**

This brief recommends systematic changes, including later school and university start times, the integration of sleep hygiene education into curricula, strict limits on late-evening activities, and the use of flexible learning models. Supporting these structural changes, systems for monitoring student well-being and educating families are also advised.

**Conclusions:**

Aligning educational schedules with biological sleep needs is a critical public health intervention. Comprehensive strategies that combine schedule adjustments, education, and support services offer the greatest benefit for student health and academic success. Future research should focus on pilot implementations and socio-economic barriers specific to Iran to ensure equitable implementation.

## Introduction

Sleep is recognized as a vital and structured process that follows a regular, cyclical pattern each night. This organized sequence of events ensures the optimal restoration of both physical and mental functions. ^1^ Indeed, sleep plays a crucial role in human psychological and physical health and is a key factor in regulating emotion, cognition, psychosocial development, and physical growth. ^2^ Inappropriate sleep patterns constitute a serious risk factor for poor physical health ^3^ and significantly diminish quality of life. ^4^ It is essential for individuals to maintain proper sleep schedules and obtain sufficient sleep, as sleep insufficiency impairs daily functioning and incurs substantial human, social, and economic costs. ^5^ Since sleep timing is a critical aspect, ^6^ sleep should occur within an appropriate temporal framework.

Healthy sleep necessitates several conditions, including adequate duration, satisfactory quality, appropriate timing, and consistent regularity. It also depends on the absence of sleep disorders. ^7^ However, healthy sleep, or sleep hygiene, often receives insufficient attention today, and school and university schedules frequently disregard student sleep patterns in their educational planning. ^8^ The scale of the issue is significant, with studies indicating that a considerable portion (e.g., over 60% in some epidemiological studies) of the student population exhibits poor sleep hygiene and insufficient sleep quantity. ^9^

In Iran, schools typically commence between 7:30 and 8:00 AM, and no national policy currently requires consideration of student sleep biology in scheduling decisions. Iranian studies have documented that a substantial proportion of students exhibit poor sleep hygiene and insufficient sleep duration, ^8^^,^^9^ and that poor sleep quality is associated with academic burnout. ^10^ However, no published research has examined the effects of delayed school start times in Iranian schools. This policy brief therefore synthesizes international evidence and proposes recommendations specifically for the Iranian educational system, while identifying critical gaps that require local investigation.

## Policy outcomes and implications

This policy brief is evidence-informed policy synthesis of the international current scientific literature on adolescent sleep, circadian rhythms, and school start times, with the objective of applying this evidence to the Iranian educational context. We prioritized evidence from authoritative policy statements (e.g., American Academy of Pediatrics, 2014), ^11^ meta-analyses, ^12^ and systematic reviews, ^13^ and integrated these with epidemiological data on sleep patterns among Iranian students. ^8^^–^^10^

A fundamental conflict exists between the rigid schedules of educational institutions and the biological clocks of students. Despite compelling evidence on adolescent sleep needs, school start times are often dictated by history and logistics, not student physiology. ^14^ This conflict is exacerbated by modern lifestyles that push bedtimes later, while long commutes and early class times force students to wake prematurely. This misalignment between educational schedules and circadian rhythms exposes students to the well-documented consequences of chronic sleep deprivation, thereby putting at risk their health, safety, and academic performance. Research has shown that sleep problems during the educational years are linked to broader psychosocial challenges, underscoring the multifaceted impact of sleep on student well-being. ^9^

The core problem lies in the direct impact of early start times on sleep duration and quality. Early classes force students to wake earlier, which, even with unchanged bedtimes, truncates total sleep time and leads to a cumulative sleep deficit. This forced sleep restriction is associated with significant issues, including academic burnout and declined academic performance. ^10, 15^ The consequences, however, extend far beyond the classroom, demonstrating measurable physiological consequences. Inadequate sleep is linked to poorer diet quality, decreased insulin sensitivity, and hyperglycemia. ^16^ The scale of this impact is staggering. In adolescents, very short sleep (<7 hours) can increase the odds of obesity by 69% (adjusted odds ratio [AOR] = 1.69; 95% CI: 1.39–2.05) and elevated waist circumference by 49% (AOR = 1.49; 95% CI: 1.28–1.73). ^17^ This study defined adolescents as individuals aged 10–19 years. This significantly elevates their long-term risk for type 2 diabetes and cardiovascular disease. While these correlational data are compelling, we must also consider confounding factors like academic stress and familial pressures.

Revising school start times is therefore an evidence-based necessity, not just an optional adjustment. It is crucial for protecting student well-being and educational productivity. The core of the issue is chronobiological: when early schedules conflict with students’ innate circadian rhythms, the resulting sleep deficit directly undermines their academic performance, mental health, and behavior. Empirical evidence demonstrates the solution. Results from a systematic review indicated that delaying school start times significantly increases students’ nightly sleep duration by at least 30 minutes, primarily due to a later wake-up time. ^18^ Also, this policy change is associated with improved school attendance, reduced tardiness, decreased daytime sleepiness, and enhanced academic performance. ^18^ These findings highlight the need to reevaluate school timing policies. It is now widely argued that delayed start times allow students to experience more and better-quality sleep, leading to reduced daytime sleepiness, improved cognitive and academic performance, ^19, 20^ and better overall physical and mental health. ^11, 12^ Successful implementation, however, requires a multi-systemic approach that considers potential conflicts with parental work schedules and the pervasive culture of intensive extracurricular commitments. In general, flexibility in the educational schedule could be proposed as a practical and acceptable alternative to a fixed later start time, provided that students are encouraged to adhere to a consistent sleep schedule. ^21^

## Actionable recommendations

Based on the analysis of policy outcomes and implications, we propose the following coordinated actions for educational policymakers and institutions.

### Mandate evidence-based start times

Consider adopting a national guideline recommending that schools aim for start times no earlier than 8:30 AM, beginning with pilot implementations in selected provinces. The American Academy of Pediatrics (2014) ^11^ explicitly recommends this threshold to allow adolescents the opportunity to achieve 8.5–9.5 hours of sleep. ^11^ This is supported by meta-analytic evidence ^12^ demonstrating that start times within the 8:30–8:59 AM window are associated with significantly longer sleep duration (effect size = 0.184, p < 0.05) and better overall sleep (effect size = 0.316, p < 0.05) compared with earlier start times.

### Enforce an evening activity curfew

Extracurricular and academic activities should be scheduled to conclude by 9:00 PM. The American Academy of Pediatrics (2014) ^11^ states that the average teenager has difficulty falling asleep before 11:00 PM and requires 8.5–9.5 hours of sleep. ^11^ Finishing activities by 9:00 PM allows approximately two hours for commuting, evening meals, hygiene, and time to wind down before sleep, a period consistent with healthy sleep hygiene principles. ^11^ This facilitates a bedtime near 11:00 PM, which is necessary to achieve the recommended 8.5–9.5 hours of sleep before an 8:30 AM start. ^11^ While direct empirical evidence validating this specific 9:00 PM cutoff in adolescent populations is currently lacking, it represents a logical derivation from established pediatric sleep recommendations and allows for the pre-sleep routines essential for healthy sleep onset.

### Integrate comprehensive sleep hygiene education

This education should include specific, practical strategies for managing digital device use before bedtime and addressing academic stress, both of which have been identified as factors influencing insufficient sleep and poor sleep timing in adolescents. ^11^ Additionally, emerging evidence suggests that maladaptive personality traits, particularly negative affectivity, disinhibition, and psychoticism, are associated with poor sleep hygiene behaviors (e.g., nighttime mobile phone use, late bedtime) and insufficient sleep duration in young adult populations. ^8^ While factors such as socioeconomic status may play a role, current evidence from adolescent-specific research, remains limited. ^12^ To support these efforts, systems for monitoring student sleep patterns and well-being should be established to allow for counseling and early intervention for those at risk of sleep deprivation or disorders. The educational message should promote healthy sleep patterns by emphasizing that sleep regularity, alongside duration, is critical for academic success and general health.

### Address social jetlag and sleep consistency

Students and faculty should be informed about the benefits of maintaining a consistent sleep-wake schedule across the entire week, including weekends, to stabilize circadian rhythms and mitigate the effects of social jetlag. A systematic review by Chaput et al. (2020) found that social jetlag is associated with adverse health outcomes, whereas weekend catch-up sleep is associated with better health in adult populations. ^13^ Although these findings need to be confirmed in adolescent cohorts, they highlight the importance of reducing circadian misalignment in students.

Additionally, we must educate families to guide lifestyle modifications that support healthy sleep at home. Finally, schools should create inherent system flexibility by exploring blended or online learning models. These formats can grant students autonomy over their schedules, allowing them to prioritize sleep without compromising academic responsibilities.

### Limitations of the analysis

While our recommendations are grounded in evidence, we acknowledge several limitations.

First, this policy brief applies evidence primarily from North American and European studies to the Iranian context. Direct empirical data on the effects of delayed school start times in Iran are currently lacking. Cultural attitudes toward sleep, parental work schedules, transportation infrastructure, school system logistics, and extracurricular activity patterns may differ substantially from the settings in which most studies were conducted. Pilot implementations and context-specific research are urgently needed before national-scale policy adoption can be recommended with confidence.

Second, the specific start time (8:30 AM) is supported by AAP policy and meta-analytic evidence, but the proposed activity cutoff (9:00 PM) is a logical derivation rather than an empirically validated threshold. Further research is needed to identify optimal scheduling parameters across different school levels and cultural contexts.

Third, our reliance on observational studies, which show correlation rather than proven causation. Future research must also address the limited data within specific socio-cultural contexts, such as Iran, to ensure these policies are equitably implemented.

## Conclusions/Discussion

Aligning educational schedules with student sleep biology is a critical public health intervention. Thoughtfully planned curricula and supportive policies can significantly improve students’ mental sharpness, overall health, and academic performance. To achieve this, the body clocks of adolescents and young adults must be a primary factor in setting school start times and daily schedules. The circadian phase delay is most pronounced during adolescence, making later school start times a critical, evidence-based intervention for middle and high school students. ^11, 12^ Research consistently shows that start times of 8:30 AM or later are associated with improved sleep duration, better academic performance, enhanced mental health, and reduced health and safety risks in this population. ^11, 12^ While delaying start times for older students is strongly supported, the impact on elementary school students is less direct. They often retain earlier start times due to logistical considerations (e.g., busing, childcare) and differing sleep biology. However, this aspect of scheduling is based on practical trade-offs rather than a robust body of comparative research. ^11, 12^ This alignment is plausibly expected to reduce long-term risk of cardiometabolic diseases, although longitudinal intervention data in the Iranian context are lacking. The most substantial benefits for student health and academic performance will likely arise from comprehensive approaches that integrate schedule adjustments, targeted education, and robust student support services.

## Data Availability

No data are associated with this article.
