# Supplementary material for: Aligning Sleep Patterns with Educational Schedules in Iran: Policy Recommendations for Enhancing Health and Academic Success
Source: F1000Res. 2026 Mar 2;14:1354. Originally published 2025 Dec 3. [Version 2] doi: 10.12688/f1000research.173727.2 (PMC12976609; doi:10.12688/f1000research.173727.2)
Supplement: Peer review history

# Reviewer report: Reviewer response for version 2

**DOI:** 10.5256/f1000research.196992.r464206

**Published:** 2026-03-14

**Authors:** Atanu Kumar Pati

**Affiliations:** 1 Kalinga Institute of Social Sciences, Bhubaneswar, Odisha, India

1. The title of the revised manuscript looks appropriate. However, to make it closer to the domain of chronobiology, the title should read somewhat like this: “Circadian Alignment of School Schedules in Iran: Implications for Student Health and Achievement,” or maybe “Circadian Misalignment Between School Schedules and Adolescent Sleep in Iran: Policy Implications for Health and Academic Performance.”
2. The recommended opening and closing times for the Iranian Schools are not convincing. Why should they end their activity at 21:00 or 22:00? To the best of my knowledge, the Tehran International School opens at 07:00 and closes at 17:00. Another School, Iranzamin School, opens at 08:00 and closes at 15:00.
3. Further, 1 MIN READ (May 13, 2025) published in IRANWIRE reveals that Iran’s Education Ministry announced that Iranian schools should begin at 06:00 in order to implement energy-saving measures. It further added that a single-shift school should operate between 06:00 and 13:00. In a 2-shift school, the first shift and the second shift should operate between 06:00-09:30, and 10:00-13:00, respectively.
4. In light of this scenario the recommended policy looks challenging for implementation. The authors did not discuss these aspects adequately.
5. The authors also did not discuss adequately the possible effect of school timings on the adolescent students with different chronotypes.
6. The text of the policy brief in its revised version is iterative in many places. The authors should revise the text of the policy brief. The literature review also appears to be inadequate. They should have given examples of the countries where the delay of school start time has been implemented. It has also been known that both too little and too much sleep are harmful.
7. Having said all this, the attempt of the authors to frame a policy brief addressing Circadian Misalignment Between School Schedules and Adolescent Sleep in Iran is worthwhile.

Does the paper provide a comprehensive overview of the policy and the context of its implementation in a way which is accessible to a general reader?

Partly

Is the discussion on the implications clearly and accurately presented and does it cite the current literature?

Partly

Are the recommendations made clear, balanced, and justified on the basis of the presented arguments?

No

Reviewer Expertise:

Chronobiology

I confirm that I have read this submission and believe that I have an appropriate level of expertise to state that I do not consider it to be of an acceptable scientific standard, for reasons outlined above.

# Reviewer report: Reviewer response for version 2

**DOI:** 10.5256/f1000research.196992.r463720

**Published:** 2026-03-10

**Authors:** Mikaela Irene Fudolig

**Affiliations:** 1 School of Mathematical Sciences, The University of Adelaide School of Mathematical Sciences, Adelaide, South Australia, Australia

The revised version addressed my biggest concerns with the first version.

Does the paper provide a comprehensive overview of the policy and the context of its implementation in a way which is accessible to a general reader?

Partly

Is the discussion on the implications clearly and accurately presented and does it cite the current literature?

Partly

Are the recommendations made clear, balanced, and justified on the basis of the presented arguments?

Partly

Reviewer Expertise:

computational social science

I confirm that I have read this submission and believe that I have an appropriate level of expertise to confirm that it is of an acceptable scientific standard.

# Reviewer report: Reviewer response for version 2

**DOI:** 10.5256/f1000research.196992.r463721

**Published:** 2026-03-04

**Authors:** Wissem Dhahbi

**Affiliations:** 1 University of Jendouba,, Kef, Tunisia

The revised manuscript has been significantly strengthened through the incorporation of specific evidence and structural adjustments. The authors have successfully transitioned the brief from a generalized discussion to a context-specific analysis focused on the Iranian educational system. The inclusion of empirical thresholds for school start times and the clarification of statistical data regarding health risks have addressed previous concerns regarding scientific rigor. The intellectual framework now effectively bridges chronobiological theory with actionable national policy recommendations.

Does the paper provide a comprehensive overview of the policy and the context of its implementation in a way which is accessible to a general reader?

Partly

Is the discussion on the implications clearly and accurately presented and does it cite the current literature?

Partly

Are the recommendations made clear, balanced, and justified on the basis of the presented arguments?

Partly

Reviewer Expertise:

Sports medicine and Reahbilitation

I confirm that I have read this submission and believe that I have an appropriate level of expertise to confirm that it is of an acceptable scientific standard.

# Reviewer report: Reviewer response for version 1

**DOI:** 10.5256/f1000research.191566.r449459

**Published:** 2026-01-12

**Authors:** Wissem Dhahbi

**Affiliations:** 1 University of Jendouba,, Kef, Tunisia

General Comments

 This policy brief addresses a relevant topic in chronobiology and public health: the misalignment between educational schedules and the circadian rhythms of adolescents and young adults. While the premise is supported by general consensus in sleep medicine, the manuscript in its current form suffers from significant gaps in scientific rigor, referencing, and specificity.

 Major Weaknesses:

 Lack of Evidence-Based Justification for Specific Metrics: The authors propose specific policy changes, such as a start time of "8:30 AM" and an activity cutoff of "9:00–10:00 PM". However, the text fails to provide the specific chronobiological data or literature that identifies these exact timestamps as the optimal thresholds. Without citing data on sleep latency or commute times, these numbers appear arbitrary.

 1

 Ambiguity in Scope and Methodology: The authors state the brief is based on a "synthesis and analysis of the current scientific literature". However, the methodology for this synthesis is opaque. Furthermore, the scope is inconsistent; the recommendations read as global/universal, yet the limitations section abruptly introduces "specific socio-cultural contexts, such as Iran", suggesting a regional focus that is not reflected in the title or introduction.

 1

 Reference and Formatting Errors: The reference list is incomplete, with References 1 and 2 appearing as empty placeholders. This is a fundamental lapse in academic presentation.

 Minor Weaknesses:

 Repetitive Phrasing: The text frequently reiterates the link between sleep and health without deepening the argument or introducing new variables.

 Over-reliance on Odds Ratios without Context: Statistics regarding obesity risks (69%) are presented without sufficient context regarding the population, age group, or study design from which they were derived.

 Specific Comments

 Abstract

 Page 1, Paragraph 2 (Policy and implications): The text states, "A systematic review of the literature confirms that delaying start times is an effective intervention". You must specify which systematic review is being referred to here. A policy brief must be precise in its attribution of evidence.

 Introduction

 Page 3, Paragraph 1: The statement "sleep hygiene, receives insufficient attention today" is a generalization. This requires a citation or a qualification (e.g., "in educational policy planning") to be scientifically valid.

 Page 3, Paragraph 1: The authors claim a "considerable portion of the student population exhibits poor sleep hygiene". Quantify "considerable" using prevalence data from recent epidemiological studies to strengthen the impact of the problem statement.

 Policy Outcomes and Implications

 Page 3, Paragraph 2: The manuscript argues that school start times are "dictated by history and logistics, not student physiology". While logically sound, this argument requires citations regarding the historical development of school schedules to differentiate between assumption and fact.

 Page 3, Paragraph 3: The claim "In adolescents, very short sleep can increase the odds of obesity by 69% and elevated waist circumference by 49%" needs immediate context. Are these Adjusted Odds Ratios (AOR)? Which specific age bracket defines "adolescents" in the cited study?

 Page 3, Paragraph 3: The sentence "Results from a systematic review indicated that delaying school start times significantly increases students' nightly sleep duration by at least 30 minutes" is missing its citation marker in the text flow. It appears the authors intended to cite Reference 14 here, but the link is not explicit.

 Actionable Recommendations

 Page 4, Paragraph 1: The recommendation "classes not begin before 8:30 AM" is scientifically weak without justification. Why 8:30 AM? Why not 9:00 AM or 10:00 AM? The American Academy of Pediatrics and other bodies have issued specific statements; the authors should reference such guidelines or specific chronotype data (e.g., shifts in melatonin secretion onset) to justify this exact time.

 Page 4, Paragraph 1: The recommendation for activities to conclude by "9:00-10:00 PM" fails to account for commute time, post-activity hygiene, and meal consumption. If a student finishes at 10:00 PM, they may not sleep until midnight, potentially negating the benefit of an 8:30 AM start. This recommendation needs a more rigorous calculation of "time-to-bed" mechanics.

 Page 4, Paragraph 1: The mention of "personality traits" as a factor in sleep hygiene adherence seems to specifically highlight the authors' own work (Reference 11). Ensure this is balanced with broader literature on behavioral barriers to sleep hygiene (e.g., socioeconomic status, blue light exposure).

 1

 Limitations

 Page 4, Paragraph 3: The sudden mention of "Iran" is jarring. If this policy brief is intended for the Iranian educational system, the title and introduction must reflect that specificity (e.g., "Policy Recommendations for the Iranian Educational System"). If it is global, this limitation should be framed more broadly regarding non-Western socio-cultural contexts.

 References

 Page 4, References Section: References 1 and 2 are missing entirely. This indicates a lack of final proofreading before submission.

 Page 4, Reference 11: The citation year is listed as "2025". Ensure this is correct and already published/in-press, as future-dated citations can be problematic if the volume/issue is not finalized.

 Scientific Quality and Methodology

 Logic: The logical flow is generally sound (Problem -> Evidence -> Solution), but the "Solution" phase lacks the granular data required for implementation.

 Statistics: The manuscript relies on secondary reporting of statistics (e.g., odds ratios). No original statistical analysis is performed, which is acceptable for a Policy Brief, provided the sources are cited accurately. However, the lack of confidence intervals for the reported percentages weakens the statistical presentation.

 Writing: The writing style is functional but repetitive. The authors acknowledge using AI for language editing. While the grammar is generally correct, the text lacks the nuance of expert human argumentation in the "Recommendations" section.

Does the paper provide a comprehensive overview of the policy and the context of its implementation in a way which is accessible to a general reader?

Partly

Is the discussion on the implications clearly and accurately presented and does it cite the current literature?

Partly

Are the recommendations made clear, balanced, and justified on the basis of the presented arguments?

Partly

Reviewer Expertise:

Sports medicine and Reahbilitation

I confirm that I have read this submission and believe that I have an appropriate level of expertise to state that I do not consider it to be of an acceptable scientific standard, for reasons outlined above.

# Reviewer report: Reviewer response for version 1

**DOI:** 10.5256/f1000research.191566.r441527

**Published:** 2026-01-12

**Authors:** Mikaela Irene Fudolig

**Affiliations:** 1 School of Mathematical Sciences, The University of Adelaide School of Mathematical Sciences, Adelaide, South Australia, Australia

The authors make a good case for changing class schedules to address the lack of sleep among students. Literature is cited for the link between delaying the start time of school and enhanced academic performance and decreased sleepiness. While the deleterious effects of a lack of sleep on health are mentioned, the correlation between delayed school start times and improved health measures are not included ("It is now widely argued that delayed start times...leading to...better overall physical and mental health" has no associated references). If this is due to a lack of studies in this matter, this should be mentioned.

 The brief lacks justification for the specifics in the actionable recommendations. The authors propose that classes should not begin before 8:30AM and that school activities should conclude by 9-10PM. However, there is no mention in the brief why these times were chosen. To justify these schedules, we need to know the time that students (generally) go to bed and get up from bed that are optimal for them. To illustrate: if students are shown to need to wake up by 9AM to feel rested, then simply mandating school to not start before 8:30AM will not fix the problem. In this case, perhaps a compressed school schedule to allow for a later start time, or one expanded to include weekends (perhaps extracurriculars may be moved to weekends to allow for a longer time for academic subjects during the weekdays), may be more beneficial. Table 4 in Reference 9 of the brief shows that the children in their sample had a mean usual wake time of 9.54 (I assume this is in hours?) on weekends, which is way beyond the 8:30AM proposed. My colleagues and I have also studied sleep patterns among university students and found that weekend sleep patterns are different from those during vacation, indicating weekend compensation due to weekday sleep debt (Fudolig, et al. "Collective sleep and activity patterns of college students from wearable devices," *npj Complexity* **2**, 32 (2025)). Whether something similar has been observed for school-age children should also be discussed in the brief if the authors wish to stick with suggesting a start time that is earlier than the weekend wake times in the study they cited.

 The authors also did not mention how the policy would address if and how sleep times vary with age. Do young children sleep the same as adolescents? How will this impact the policy?

 Overall, I was convinced by the authors that sleep timing and duration of students must be taken into account when developing policy in order to improve well-being and academic achievement. However, I believe the authors have to support their specific actionable recommendations with more studies.

Does the paper provide a comprehensive overview of the policy and the context of its implementation in a way which is accessible to a general reader?

Partly

Is the discussion on the implications clearly and accurately presented and does it cite the current literature?

Partly

Are the recommendations made clear, balanced, and justified on the basis of the presented arguments?

Partly

Reviewer Expertise:

computational social science

I confirm that I have read this submission and believe that I have an appropriate level of expertise to confirm that it is of an acceptable scientific standard, however I have significant reservations, as outlined above.
